# Should dispersers be fast learners? Modeling the role of cognition in dispersal syndromes

**DOI:** 10.1002/ece3.8145

**Published:** 2021-09-22

**Authors:** Jannis Liedtke, Lutz Fromhage

**Affiliations:** ^1^ Department of Biological and Environmental Science University of Jyvaskyla Jyvaskyla Finland; ^2^ Institute of Zoology University of Hamburg Hamburg Germany; ^3^ BioConsult SH GmbH & Co., KG Husum Germany

**Keywords:** behavior syndromes, cognition, cognitive styles, invasion, life history, pace of life

## Abstract

Both cognitive abilities and dispersal tendencies can vary strongly between individuals. Since cognitive abilities may help dealing with unknown circumstances, it is conceivable that dispersers may rely more heavily on learning abilities than residents. However, cognitive abilities are costly and leaving a familiar place might result in losing the advantage of having learned to deal with local conditions. Thus, individuals which invested in learning to cope with local conditions may be better off staying at their natal place. In order to disentangle the complex relationship between dispersal and learning abilities, we implemented individual‐based simulations. By allowing for developmental plasticity, individuals could either become a 'resident' or 'dispersal' cognitive phenotype. The model showed that in general residents have higher learning abilities than dispersers. Dispersers evolve higher learning ability than residents when dispersers have long life spans and when dispersal occurs either early or late in life, thereby maximizing the time in one habitat patch. Time is crucial here, because the longer an individual resides in a location where it can use its learned knowledge or behavior, the more often it profits from it and thus eventually obtains a net benefit from its investment into learning. Both, longevity and the timing of dispersal within lifecycles determine the time individuals have to recoup that investment and thus crucially influence this correlation. We therefore suggest that species' life history will strongly impact the expected cognitive abilities of dispersers, relative to their resident conspecifics, and that cognitive abilities might be an integral part of dispersal syndromes.

## Graphical abstract

Using individual‐based simulations, we show that dispersal and learning abilities can be either positively or negatively correlated, depending critically on the time span individuals have to recoup their investment into learning. Both longevity and timing of dispersal crucially determine this time span. We thus conclude that these life‐history traits crucially determine this correlation and suggest that in order to better understand animal dispersal patterns and success one should take the cognitive abilities of investigated species into account.

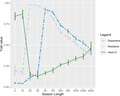

## 1 INTRODUCTION

There is accumulating evidence that dispersing individuals are often a nonrandom subset of their source population. For example, under intraspecific competition it is assumed that weaker individuals are driven out and thus more likely to disperse (Bonte & de la Pena, 2009). However, under the perspective of inclusive fitness, it has been suggested that kin competition can lead to stronger and more competitive individuals leaving their natal place and compete with nonkin in new patches (Bonte & de la Pena, 2009; Gyllenberg et al., 2008). Dispersal is a complex process which can be divided into three phases: departure, transfer, and settlement (Bowler & Benton, 2005). Because all three phases involve challenges that differ from the day‐to‐day challenges an individual faces when staying at its natal place, dispersing individuals may adjust their phenotypic traits accordingly. When multiple such traits are shaped in concert, this is called a 'dispersal syndrome' (Clobert et al., 2009; Cote & Clobert, 2012; Legrand et al., 2016; Sih et al., 2004). Such 'super dispersers' can have different morphological features to facilitate movement (e.g., wing or body size; reviewed in Bonte et al., 2012), increased fat reserves (O’Riain et al., 1996), or may be expected to have different behavioral responses to optimize performance in new environments (Sih et al., 2004), for example, toward predators (compare Geffroy et al., 2020), conspecifics (Toor et al., 2020), or unknown objects (compare Mettke‐Hofmann et al., 2005).

Species differ quite strongly in their cognitive abilities, which are often positively correlated with longevity (e.g., Deaner et al., 2003; Sol, 2009). However, even short‐living species are showing astonishing cognitive abilities, ranging from associative learning in nematodes (Sasakura & Mori, 2013), reversal learning in spiders (Liedtke & Schneider, 2014), to social learning and teaching in insects (Alem et al., 2016). In recent years, it has become clear that individuals of the same species differ in their cognitive abilities (Boogert et al., 2018; Cauchoix et al., 2018; Liedtke & Fromhage, 2019a), raising the question whether these between‐individual differences may correlate with other traits such as dispersal, thus potentially being part of a 'dispersal syndrome' as described above.

Cognitive abilities may be beneficial during all three dispersal stages as they allow to, for example, gather information about and to compare unknown habitats (Clobert et al., 2009; Cote & Clobert, 2012; Delgado et al., 2014; Edelaar et al., 2017; Maspons et al., 2019; McNamara & Dall, 2011). Yet, there is not much known about how individual differences in cognitive abilities may relate to differences in dispersal tendencies. Furthermore, cognitive abilities, in general, are expensive (e.g., metabolic costs, Niven & Laughlin, 2008) and when dispersers settle in an environment in which these abilities are less needed, the costs may outweigh their benefits. Thus, under some circumstances it may be better for dispersers to have lower cognitive abilities in order to save these costs. It is therefore conceivable that cognitive abilities can be adjusted for dispersal during development, thus being an integral part of 'dispersal syndromes'.

In another study, we showed that dispersal tendency and learning abilities can evolve in a correlated manner in a metapopulation setting (Liedtke & Fromhage, 2021), where distinct trait combinations emerged across different habitat (patch) types. That study, however, made the simplifying assumption that an individual's learning abilities were fully determined by its genotype, regardless of whether it dispersed or not. This essentially meant that different traits could not influence each other during development, thus precluding the evolution of an optionally expressed “dispersal syndrome” involving multiple traits. The present study is designed to relax this constraint. To this end, we model the evolution of a genotypic strategy that can encode two independently evolving alternative phenotypes—a “resident” and a “disperser” phenotype which are expressed in these respective contexts. Specifically, we assume that each individual faces a developmental switch with two options: either it expresses its genotypically encoded “resident” phenotype and is then destined not to disperse; or it expresses its genotypically encoded “disperser” phenotype and is then destined to disperse. These adjustments are irreversible and cannot be changed during an individuals' lifetime. This modeling approach seems especially appropriate for species where residents and dispersers differ in traits linked to dispersal, even among individuals originating from the same patch. Wing dimorphism, for example, is commonly found in insects, with large‐winged (macropterous) individuals constituting the dispersal morph (Roff, 1986), which also may be linked to metabolism specialization (Van Belleghem & Hendrickx, 2014). In naked mole rats, young males can develop into a dispersing morph, including increased fat reserves and behavior adaptations, presumptively for increasing outbreeding (O’Riain et al., 1996). In common lizards, dispersal dimorphism is responsive to environmental change (e.g., fragmentation, Cote et al., 2017). Furthermore, whether the investment in cognitive abilities can be recouped, and thus can be adaptive, crucially depends on how much time animals have available to use these abilities (Liedtke & Fromhage, 2019b and refs. therein). Longevity and timing of dispersal crucially influence the duration of this recouping phase by determining how long an individual will reside in one patch. We therefore investigate the effects of longevity and timing of dispersal on the interplay between cognitive abilities and dispersal. This allows us to assess the role of life‐history traits in shaping dispersal syndromes. Because dispersal syndromes have mostly been documented for relatively short‐lived species, we note that those are the species for which our present modeling approach may be most likely to be relevant. On the other hand, we see no compelling theoretical reason why the processes studied in our model should not occur, in a qualitatively similar form, in long‐lived species too.

## 2 METHODS

This model is an extension of a previous model (Liedtke & Fromhage, 2021) about the joint evolution of cognitive styles and dispersal tendencies. The description of methods is therefore largely identical, except for the implementation of developmental plasticity (see below) and the exclusion of predation (for simplicity).

We implemented a metapopulation setting with *N*_Patches_ habitat patches, which are connected through random global dispersal, that is, individuals have the same chance of reaching any of the *N*_Patches_ patches when dispersing (list of abbreviations see Table 1). Carrying capacity of each patch is set to *N*_Individuals_ and three traits are allowed to evolve independently for *N*_Generations_: learning ability *L*, exploration tendency *E* (i.e., explorative foraging within patch), and dispersal tendency *D*. All three traits are continuous with values between 0 and 1. At the end of each generation, individuals reproduce asexually in proportion to their fitness. Fitness of individuals is specified by the amount of resources they obtain during their lifetime. We assume an 'income breeder' system where individuals may reproduce independently of their survival until the end of season.

**TABLE 1:** Abbreviations

Abbreviation	Description
*A_Ri_*	Abundance of different resource types
*C_Ri_*	Detectability of resource type *i*
*D*	Dispersal tendency
*E_D_*	Exploration tendency for dispersers
*E_R_*	Exploration tendency for residents
*F*	Reproductive success (fecundity)
*H_Ri_*	Handling time of resource type *i*
*L_D_*	Learning ability for dispersers
*L_R_*	Learning ability for residents
*M*	Mortality risk
*N*_Generations_	Number of generations
*N*_Individuals_	Carrying capacity of *Pi*
*N*_Patches_	Number of patches
*N_Ri_*	Maximum number of resource items per type per individual
*P_i_*	Patch number *i*
*R_i_*	Resource type *i*
*R*max*_i_*	Maximum total amount of resources *R*max_i_ in a given patch
*T*_after_	Length of season after dispersal
*T*_before_	Length of season before dispersal
*V_Ri_*	Value of resource type *i*
*V*_Total_	Sum of value of all resources collected by a given individual
Α	Cost coefficient of learning
*Μ*	Mutation probability
Ф	Competition factor

The length of a season (=generation) is defined by the number of days before dispersal *T*_before_, plus the number of days after dispersal *T*_after_. For simplicity, all dispersers will disperse at the same moment and dispersal does not consume any time. Cost of dispersal is implemented as mortality risk *M* during dispersal.

The lifecycle of individuals proceeds in four phases: (1) time before dispersal in which they can collect resources; (2) potential dispersal event, that is, moving with some probability from one patch to another, with a mortality risk defined by *M*; (3) time after dispersal for collecting resources; (4) asexual reproduction followed by death. After the last phase, a new generation starts with offspring generated by the parent generation.

### 2.1 Development

Because optimal traits values (*L* and *E*) may differ for residents and dispersers, we allow for developmental plasticity, by letting the expression of *L* and *E* to be conditional on dispersal. Whether or not an individual will disperse is determined at the very beginning of its life, depending on its trait value *D* and a threshold value between 0 and 1 randomly drawn from a uniform distribution. When the individual's dispersal tendency (*D*) is higher than that threshold, the individual will disperse later in life; otherwise, it will stay in its natal place. Depending on the now determined fate of individuals as either a resident or a disperser, they will develop different phenotypes, which are encoded by two independent loci: one locus determines the learning abilities for residents (*L_R_*) and the other for dispersers (*L_D_*). Similarly, there are two loci for exploration tendency with *E_R_* encoding exploration for residents and *E_D_* for dispersers. If the individual will be a resident, it expresses *L_R_* and *E_R_*; if it will disperse, it expresses *L_D_* and *E_D_*, respectively. Traits do not change at the time of dispersal, but instead remain constant throughout an individual's life. Each locus underwent independent mutation as described below and thus could evolve independently.

### 2.2 Environment

The environment of a patch is defined by its patch size *N*_Individuals_ and the abundance (*A_Ri_*) of different resource types *R_i_*. Abundances are defined as the maximal number of resource items of type *R_i_* which an individual can encounter in a given period of time (see below). Patches can differ in their composition of available resources. Furthermore, resources are defined by their value *V_Ri_* in terms of increasing fitness, their handling time *H_Ri,_* that is, how long individuals need to handle them before they can obtain their value, and their detectability *C_Ri_*, that is, how easy they are to find. For simplicity, we implemented simulations with two, equally frequent, patch types.

### 2.3 Learning

Learning is implemented as a reduction in handling time (*H_Ri_*) of resources due to gaining experience with specific resource types, reflecting the idea that some feeding techniques need to be practiced repeatedly before succeeding (such as tool use in primates (Boesch et al., 2019) and birds (Kenward et al., 2006), or hunting techniques in dolphins (Guinet & Bouvier, 1995)). Up to ten different resource types are implemented, with *R*_1_ being a simple‐to‐access resource whose handling requires no learning. *R*_2_ to *R*_10_ are resources for which individuals need experience before they can exploit them. Therefore, individuals get better at exploiting resource items of type *R*_2_ through *R*_10_ with time. Learning experience with specific resource types can be carried over to new settlement patches if (and only if) dispersers will find the same resource type in the new patch. A detailed description of how learning was calculated follows below.

### 2.4 Resource intake

First, we calculate the maximum number of resource items per type (*R_i_*) an individual can collect before dispersal, by multiplying the abundances (*A_Ri_*) in patch *P_i_* with the time it has to do so (i.e., *T*_before_). Based on the results found in a previous study (Liedtke & Fromhage, 2019a), we assumed that individuals will explore their surroundings at least every second time step. Whether individuals would also move in the other timesteps depends on their exploration tendency (*E_i_*). The higher its *E_i,_* the more likely an individual moves and encounters further resources, such that its maximum number of resource items of type *R_i_* is given by.

(1) $$N_{\mathrm{Ri}}=A_{\mathrm{Ri}}*T_{\text{before}}*(1+E_{i})$$

This formulation implies that individuals with *E_i_* = 0 move at a slow pace and gain maximally half of what individuals with *E_i_* = 1 gain.

Next, we take into account the individuals' exploration tendency *E_i_* and the detectability of resource types *C_Ri_*. We assume that the faster an individual explores, the less thoroughly it can search; and the harder the items are to detect (i.e., low *C_Ri_*), the less likely the individual will find a resource. This changes the calculation of collected resources as:

(2) $$N_{\mathrm{Ri}}^{\prime }=N_{\mathrm{Ri}}*\left(1‐\left(1‐C_{\mathrm{Ri}}\right)*E_{i}\right)$$

Thereafter, we take into account each individual's efficiency of handling resources as influenced by its learning speed *L* and the number of resource items collected, that is, how much experience it gained with a specific resource type. This changes the calculation of collected resources as:

(3) $$N_{\mathrm{Ri}}^{\prime \prime }=\sum_{j=1}^{\text{round}(N_{\mathrm{Ri}}^{\prime })}\text{max}\left(0,1‐\frac{\sqrt{H_{\mathrm{Ri}}‐1}}{\sqrt{j}*L}\right)$$

where *H_Ri_* is the handling time of *R_i_*. This formula was selected because it describes a decline of handling time at a decelerating rate. This functional shape appears biologically plausible because perfection may often be difficult to reach, which may slow progress down once more progress has been made. Note that resources with high *H* need to be handled multiple times before they can be exploited by a given individual.

Finally, we take into account intraspecific competition over resources within a patch. First, we estimate the maximum total amount of resources *R*max_i_ potentially collected by all individuals in a given patch, adjusted by a competition factor Ф that controls the severity of the competition:

(4) $$R{\text{max}}_{i}=T_{\text{before}}*A_{\mathrm{Ri}}*N_{\text{Individual}}/\Phi$$

Then, we divide this by the sum of resources collected by all individuals as estimated by Equation 3, to obtain the ratio $R\max_{i}/\sum N_{\mathrm{Ri}}^{\prime \prime }$. If this ratio is <1, then the focal resource type is completely depleted, and the share collected per individual is reduced by competition as:

(5) $$N_{\mathrm{Ri}}^{\prime \prime \prime }=N_{\mathrm{Ri}}^{\prime \prime }*R\max_{i}/\sum N_{\mathrm{Ri}}^{\prime \prime }$$

For example, if (according to precompetition calculations) resource type *R*_2_ was collected 10 times more often than its *R*max*_i_* value for this patch, then for every individual in this patch its amount of collected *R*_2_ items is multiplied by 0.1.

### 2.5 Dispersal

After this foraging phase, individuals could disperse to a randomly chosen patch. An individual's decision to disperse or not was determined at the beginning of its life as described above. Due to the stochasticity of this process some patches may have lower, others higher numbers of individuals after the dispersal phase. Dispersal costs are implemented as mortality risk *M* which was set to 0.01 in all cases. Whenever an individual attempts to disperse, a random number between 0 and 1 is drawn from an uniform distribution. If this number is lower than *M*, the individual dies; otherwise, it successfully disperses.

After the dispersal phase, surviving individuals are allowed to collect resources again. Resource intake and competition are calculated as in the predispersal phase (Equations (1), (2), (3), (4), (5)) with the only difference being that the duration of the postdispersal phase is defined by *T*_after_. Note that dispersers may need to learn how to gain hard‐to‐access resources again, if a different resource type is found in the settlement patch. Since residents stay in their natal patch, they need to learn only one type of hard‐to‐access resource type (with given settings presented in this study).

### 2.6 Reproduction

After estimating the total resource income of all individuals, reproductive success (fecundity) is calculated as:

(6) $$F=V_{\text{Total}}*\left(1‐L*\alpha \right)$$

where *L* is an individual's learning ability, *α* a cost coefficient which specifies the cost of learning, and *V*_Total_ is the sum of value of all resources collected by this individual. We do not include any explicit cost of *E* because costs of exploration are implicit in the risks of overlooking resources. The next generation is recruited in each patch independently, by using local individuals' *F* value as the independent sampling probability. Thus, the higher *F* of a focal individual is compared to all other individuals in the same patch, the more likely it contributes offspring to the total *N*_Individuals_.

### 2.7 Mutation

Mutation probabilities for all three traits (*L*, *E*, *D*) are set to *μ* = 0.1. Traits evolve independently and new values are chosen randomly from a normal distribution with the parental trait value as mean and *SD* of 0.1.

### 2.8 Extinction

To increase the incentive to disperse, it is common practice in modeling studies to implement random extinction of patches (Poethke et al., 2003). We do so by erasing, with a given frequency, all individuals of a randomly selected patch at the end of a generation. The empty patch can then only be recolonized by emigrants from other patches within the metapopulation.

### 2.9 Initialization

Initially, we heuristically explored the parameter space in order to find parameter settings allowing the evolution of different cognitive styles which can coexist both within (compare Liedtke & Fromhage, 2019a) and between patches.

For simplicity, the main results presented here are derived from simulations in which detectabilities of resources (*C_Ri_*) were the same and thus *E* of all individuals evolved to be similar. This allows us to concentrate on the effects of learning abilities on dispersal and vice versa, which is our main interest here.

Parameter settings for each of the presented simulation sets are given in Tables 2 and 3. All simulations presented were replicated 10 times with identical parameter settings. All replicate runs produced qualitatively similar results.

**TABLE 2:** Parameter settings for simulation presented in Figure 1 main text

Abbreviation	Description	Parameter setting
*A_Ri_*	Abundance of different resource types	In patch type 1: R1 = 1, R2 = 5; In patch type 2: R1 = 1, R3 = 5
*C_Ri_*	Detectability of resource type *i*	R1 = R2 = R3 = 0.5
*H_Ri_*	Handling time of resource type *i*	R1 = 1, R2 = R3 = 300
*M*	Mortality risk	0.01
*N*_Generations_	Number of generations	300
*N*_Individuals_	Carrying capacity of *Pi*	100
*N*_Patches_	Number of patches	12
*T*_after_	Length of season after dispersal	2, 4, 10, 15, 25, 50, 150, 250, 500, 1,000, 2,000
*T*_before_	Length of season before dispersal	2, 4, 10, 15, 25, 50, 150, 250, 500, 1,000, 2,000
*V_Ri_*	Value of resource type *i*	R1 = 1, R2 = R3 = 10
*Α*	Cost coefficient of learning	1.4
*Μ*	Mutation probability	0.1
Ф	Competition factor	2
*EX_freq_*	Extinction frequency (every × generation)	2
*EX_N_*	Number of patches getting erased every *EX_freq_* generation	1

**TABLE 3:** Parameter settings for simulation presented in Figure 2 main text

Abbreviation	Description	Parameter setting
*A_Ri_*	Abundance of different resource types	In patch type 1: R1 = 1, R2 = 5, R3 = 0 In patch type 2: R1 = 1, R2 = 0, R3 = 5
*C_Ri_*	Detectability of resource type *i*	R1 = R2 = R3 = 0.5
*H_Ri_*	Handling time of resource type *i*	R1 = 1, R2 = R3 = 150
*M*	Mortality risk	0.01
*N*_Generations_	Number of generations	500
*N*_Individuals_	Carrying capacity of *Pi*	100
*N*_Patches_	Number of patches	12
*T*_after_	Length of season after dispersal	2, 10, 18
*T*_before_	Length of season before dispersal	18, 10, 2
*V_Ri_*	Value of resource type *i*	R1 = 1, R2 = 10
*Α*	Cost coefficient of learning	1.4
*Μ*	Mutation probability	0.1
Ф	Competition factor	6
*EX_freq_*	Extinction frequency (every × generation)	2
*EX_N_*	Number of patches getting erased every *EX_freq_* generation	1

To compare learning abilities (*L*) between residents and dispersers, we applied paired *t* tests. Separately for each season length (*S = *4 to 4,000), we used the mean values of *L* of residents and dispersers of each replicated simulation run as independent datapoints (degrees of freedom = 9).

All traits reached equilibrium—as judged by visual inspection—well within given generations numbers.

## 3 RESULTS

**FIGURE 1:**
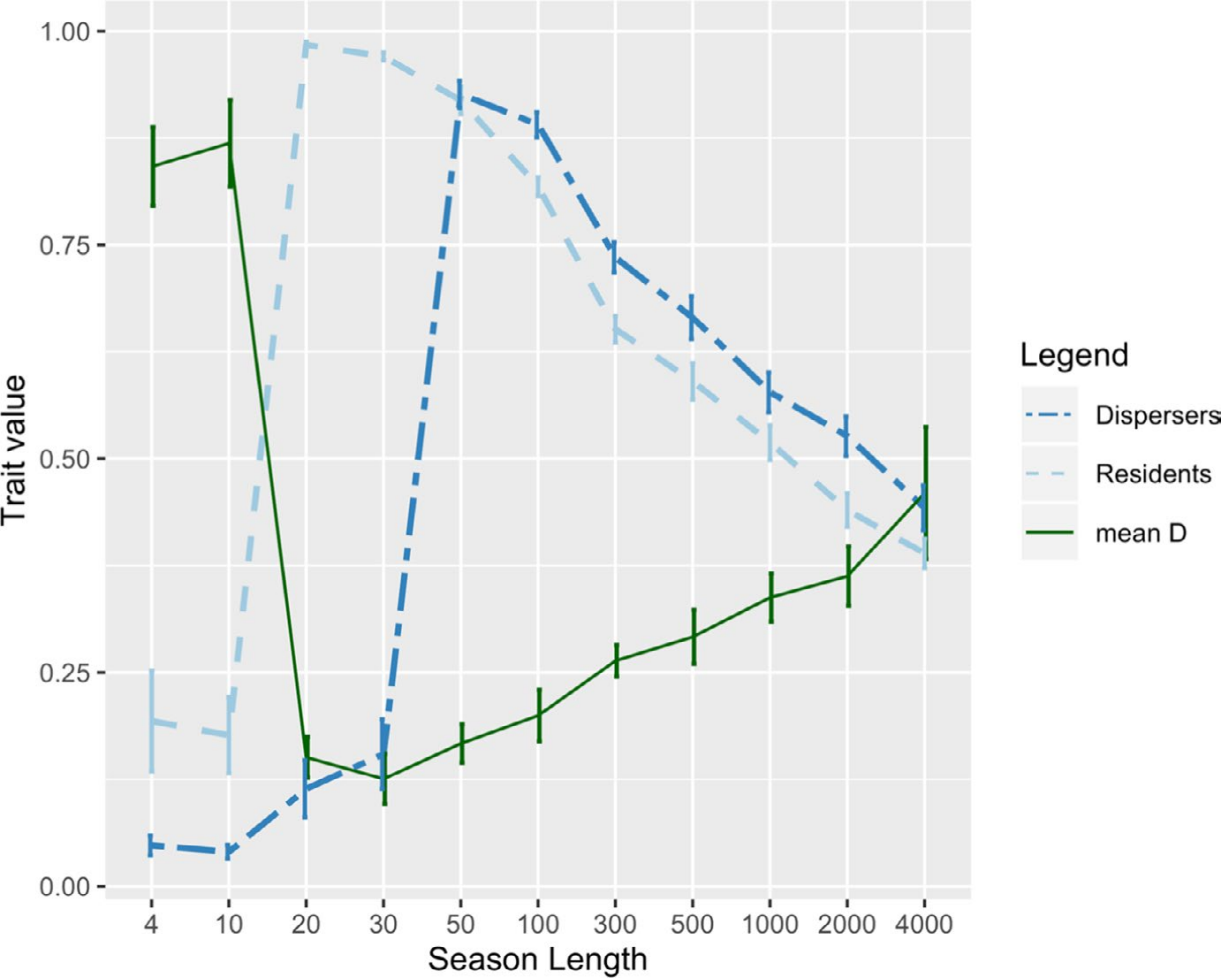
The figure shows the mean for trait values *L* (=learning speed) for residents (*L_R_*), dispersers (*L_D_*) and the population's mean dispersal tendency (mean *D*). Each point represents the mean of ten replicated simulation runs. In each run traits reached equilibrium well within given number of generations. Time before and after dispersal was equally long in all simulations (*T*_before_ = *T*_after_). For all but one simulated season length the mean differed significantly between residents and dispersers. Error bars indicate standard deviation. For season length = 50 there was no significant difference

Season length (i.e., *T*_before_ + *T*_after_; equivalent to life span) crucially determines whether dispersers had higher or lower *L* than residents (see Figure 1, Table A1). With very short life spans, individuals did not invest into higher learning speed and both *L_R_* and *L_D_* were low accordingly. However, since dispersal tendency *D* was very high, there were only very few residents present and thus selection for *L_R_* was low. Due to mutation–selection balance (Crow & Kimura, 1970), *L_R_* was pushed upwards (Figure 1, Season length (*S*) = 4 and 10), that is, closer to the value 0.5 expected for a selectively neutral trait. With slightly longer season length, residents, which by definition stayed in their birth patch their whole life, became able to exploit hard‐to‐access resources if they invested strongly into learning abilities (i.e., *L_R_*). This led to a huge increase in *L_R_* compared to learning abilities of dispersers (i.e., *L_D_*) which were unable to exploit hard‐to‐access resources within their given time (Figure 1, *S* = 20 and 30). When increasing the total season length further, also dispersers were able to exploit hard‐to‐access resources (both in their natal and new settlement patches) and invested highly into *L*. As a result, the differences between *L_R_* and *L_D_* first becomes insignificant (Figure 1, Table 2, *SL* = 50) and then, with increased *SL*, reverses direction, that is, *L_R_* becomes significantly lower than *L_D_* (Figure 1, Table 2, *S* > 50).

**FIGURE 2:**
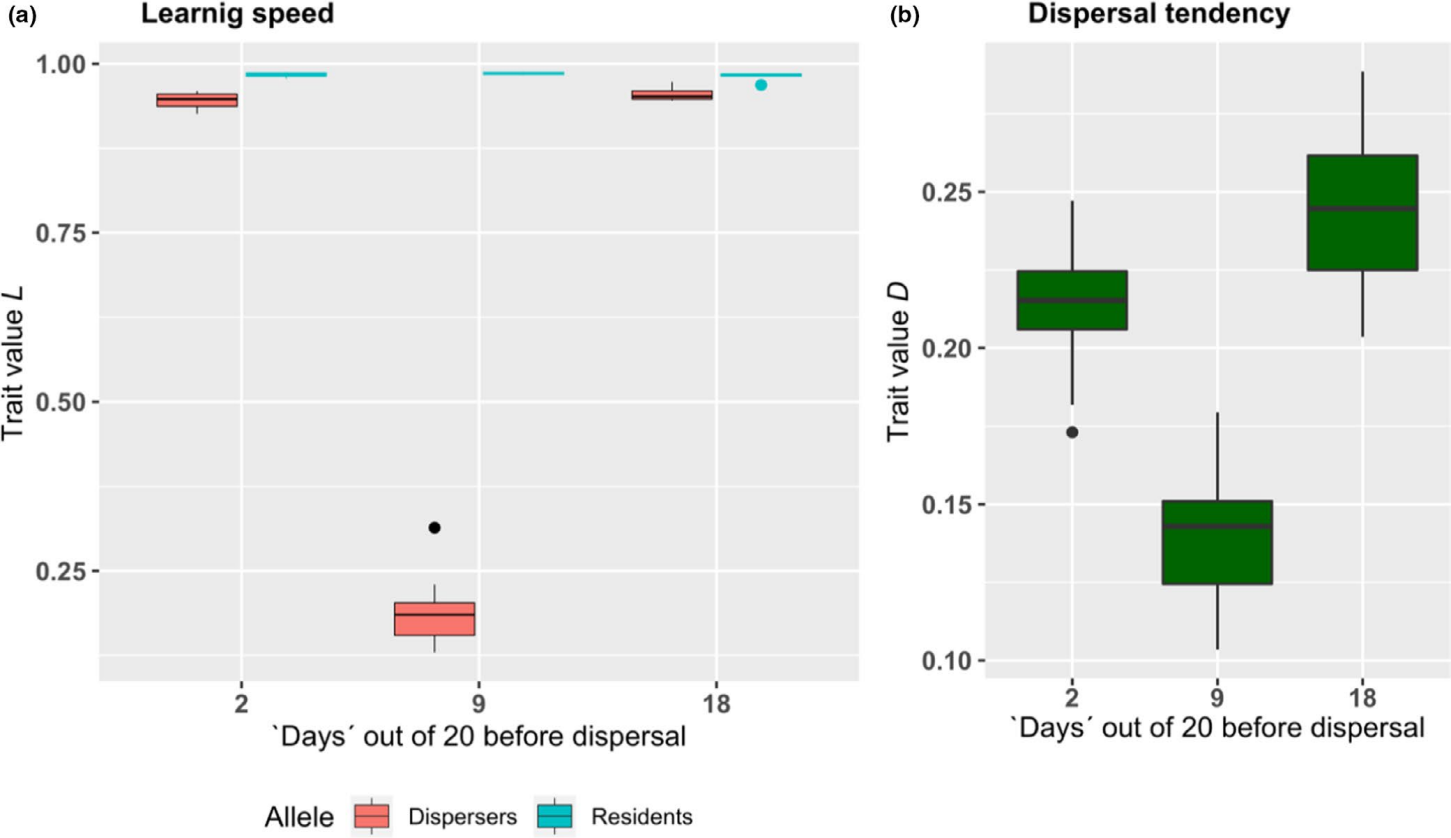
(a) Mean trait values *L* (=learning speed) for residents (*L_R_* in blue, with matching contour colours to make colours discernible despite low variances) and dispersers (*L_D_* in red). Note that these phenotypes are encoded by different loci, only one of which is expressed in any given individual. (b) Mean dispersal tendency of whole population. Boxplots represent the results of ten replicated runs where the mean of each simulation serves as one datapoint. The season length (i.e. in 'days') was 20 in total in each case but the time point of dispersal differed. On the left‐hand side dispersal was allowed after the second, in the middle after the ninth and on the right‐hand side after the 18th 'day'

Changing the timing of dispersal within the life cycle, by the parameters *T*_before_ and *T*_after_, strongly influenced the cognitive style of disperses (Figure 2a). For a given season length, when dispersal took place in the middle of life, dispersers invested little in learning because they did not have enough time to learn either at their birthplace or in the new patch. Since this reduced the competitive abilities of dispersers, dispersal costs increased, and consequently, mean dispersal tendencies decreased (Figure 2b). However, when dispersal took place either early or late in life (e.g., breeding dispersal), then dispersers had time to adapt to at least one set of local conditions, hence investing in *L* similarly to but slightly lower than residents (Figure 2a) and dispersal tendencies increased again (Figure 2b).

## 4 DISCUSSION

Our model revealed strong effects of longevity and the timing of dispersal on evolved patterns in learning abilities. Sufficient longevity was needed for the evolution of high learning abilities, with dispersers needing longer life spans than residents to be able to recoup their investment into learning. Timing of dispersal modulated the relationship between learning abilities and dispersal, by determining the time dispersers had in either location (natal or settlement patch) to recoup their investment.

Similar to a previous model (Liedtke & Fromhage, 2019a), resource composition determined whether or not different cognitive styles could coexist within the same patch. That is, it is easy to find resource compositions where either all or no individuals invest strongly in learning (not shown). Coexistence, however, depends on individuals specializing on different resource types, such that some individuals specialize on more abundant and easy‐to‐handle resources, whereas others specialize on hard‐to‐access resources with higher value. Since individuals compete over these resources, negative frequency dependence stabilizes the coexistence (for a more detailed discussion, please see Liedtke & Fromhage, 2019a). For the hard‐to‐access resources, individuals need to invest into learning speed (*L*) in order to be able to learn to exploit them within the available time (i.e., life span). With very short life spans, time is not sufficient for learning and thus no investment in *L* occurred. Once there is enough time for learning to exploit these resources, any further increase in life span leads to a reduced investment in *L* because of relaxed time pressure (i.e., individuals can reduce learning costs by learning more slowly, provided there is enough time; compare (Liedtke & Fromhage, 2019b)). This nonlinear link between life span and investment into learning speed is the underlying cause of the effect of life span on dispersal in the present model. With very short life spans, individuals do not invest in higher *L* and consequently residents and dispersers adopt similar cognitive styles with low learning abilities (Figure 1, leftmost data points). Yet, if life span is just long enough for learning to handle hard‐to‐access resources, individuals need to invest highly in *L* to exploit these resources. Crucially, only if fast‐learning individuals encounter these resources throughout their whole life, they can recoup the investment into high *L* by increasing their resource intake and reproduction. So, if hard‐to‐access resources differ between patches, and learning progress is not transferable between resource types, then dispersers are unable to exploit hard‐to‐access resources either at their natal or at the settlement patch. Therefore, dispersers cannot recoup their investment into high *L*. Consequently, individuals investing into high *L* are better off staying in their natal place, and dispersers are better off investing little into *L*, specializing on easy‐to‐access resources instead (Figure 1, center).

With increased life spans the pattern reverses because, above some minimal life span, there is sufficient time for learning to handle resources both in the natal and in the settlement patch. Meanwhile, since residents only need to learn one type of hard‐to‐access resource, they have more time to do so and can afford to learn slower and pay less cost of *L*. Accordingly, dispersers have higher *L* than residents (Figure 1, right half). With further increase in life span, also dispersers have more time to learn and thus can likewise afford to reduce their investment in *L*.

A similar effect occurs when considering the timing of dispersal within the lifecycle of a species. Dispersal early in life allows dispersers to adjust to local conditions of the settlement patch where they spend most of their life. Provided that life span is not too long (see above), this promotes the investment into *L* for dispersers, to a similar extent as in residents. Likewise, dispersal at the end of the lifecycle allows individuals to adjust to local conditions of the natal place where they spend most of their life. Again, this leads to minimal differences in *L* between residents and dispersers. If, however, dispersal takes place in the middle of life, it divides the available time in any one place in such a way as to prevent dispersers from investing in *L*. Under these circumstances, we can find different values of *L* for residents and dispersers. Since dispersers cannot compete with residents over hard‐to‐access resources, the cost of dispersal increases, and dispersal tendencies become lower. This relationship, of course, depends greatly on the species' total life span. With very short life spans, no investment in *L* is expected whereas with very long life span, as in long‐living vertebrates such as primates or parrots, even dispersal somewhere in the middle of life should allow to adjust both to the natal and the new patch.

Besides the effect of longevity on investment in learning abilities, we can observe an effect on dispersal tendencies (*D*) as well. With very short life spans, mean dispersal tendency is high (see *S* < 20, in Figure 1). Once a prolonged life span allows residents to exploit hard‐to‐access resources, the mean value of *D* drops considerably (see *S* = 20, in Figure 1). This pattern arises because, with short life spans, no one can learn to exploit hard‐to‐access resources and all individuals compete for easy‐to‐access resources. Consequently, residents have no (foraging) advantages over dispersers and thus dispersers can compete in new patches as well as in their natal patch. This results in low costs for dispersal and high dispersal rates. Yet, once life span is long enough to permit effective learning, residents have the advantage of being faster in exploiting hard‐to‐access resources and thus outcompete dispersers, which first need to learn how to handle the new hard‐to‐access resources. This increases the costs of dispersal, leading to lower mean dispersal rates. Only with longer life spans, dispersers can become similarly efficient in exploiting hard‐to‐access resources at the settlement patch and thus are able to compete with residents. At this point, dispersal rates start to increase with longevity again (*S* > 50, in Figure 1).

Comparing our present results with those of a model without developmental plasticity (Liedtke & Fromhage, 2021), we can summarize that under both approaches a correlation between learning abilities and dispersal occurs under a wide range of environmental circumstances. Note, however, that the results of both models are in no way redundant, as biologically there is a qualitative difference between a population where dispersal and learning are correlated across habitat patches (as in Liedtke & Fromhage, 2021), and a population where dispersal and learning are phenotypically correlated even across individuals that may share the same genotype (as in the present study). Moreover, the differences between dispersers and residents are clearer when they are based on developmental plasticity. The intuitive explanation for this is that plasticity allows selection to shape alternative specialized phenotypes, for a life that either involves dispersal or not (see, e.g., Roff, 1986).

Whether such plasticity is to be expected in natural systems depends on the species and, in particular, on the ecological factors that trigger dispersal. As described in the introduction, dispersal is often a conditional process. When triggering conditions occur early in life, such as conspecific density, predation pressure, or kin competition, the developmental trajectory of dispersing individuals may be adjusted accordingly. Thus, under these circumstances we suppose that cognitive abilities, like other traits, may differ substantially between residents and dispersers and, in some cases, eventually produce dispersal syndromes. By contrast, when triggering conditions occur after the developmental phase and are not predictable beforehand, for example operational sex ratio, sudden droughts, flooding, or fire events, individuals are restricted in their adjustment to dispersal. In this case, a correlation between learning abilities and dispersal can arise at the population level through local adaptation, for example, if some habitat types favor higher values in both learning ability and dispersal tendency (Liedtke & Fromhage, 2021). However, according to our simulations, such correlations tend to be less pronounced (and hence may be harder to detect empirically) than under the developmental plasticity scenario.

In conclusion, we have shown that the interplay of cognitive abilities and dispersal can be complex. In our simulations, time is a crucial determinant of whether dispersers should be fast learners to adjust quickly to new environments, or whether dispersal interferes with the ability to reap the potential benefits of learning. More generally speaking, plasticity allows individuals to adjust to local conditions which, however, induces also costs. Whether these costs can be recouped depends on how much time the dispersers have after settlement. We therefore predict that a species' life span and the timing of dispersal within the lifecycle crucially influence the correlation between dispersal and cognitive abilities, supporting other findings underlining the importance of lifecycles when considering the evolution of dispersal (e.g., Massol & Débarre, 2015).

In this study, we simulated an annual life cycle resembling short‐lived species such as insects or spiders, and parameters like longevity or duration of learning a task were implemented accordingly. Yet we expect that the general principles of the interaction between learning, dispersal, and longevity will remain the same for longer‐lived species such as vertebrates. The crucial point is how long individuals need to learn a task in relation to their available time as determined by their ecology. However, since learning speed should depend on the frequency with which similar tasks are encountered, it should also depend on the degree of environmental complexity and predictability. Thus, an interesting avenue for further research would be to investigate how life‐history traits such as life span and timing of dispersal coevolve under variation of these environmental aspects.

## CONFLICT OF INTEREST

None declared.

## AUTHOR CONTRIBUTIONS

**Jannis Liedtke:** Conceptualization (lead); data curation (lead); funding acquisition (lead); investigation (lead); methodology (lead); project administration (lead); validation (lead); writing–original draft (lead); writing–review and editing (equal). **Lutz Fromhage:** Conceptualization (supporting); data curation (supporting); funding acquisition (supporting); investigation (supporting); methodology (supporting); project administration (supporting); resources (supporting); supervision (lead); validation (supporting); writing–review and editing (equal).

### OPEN RESEARCH BADGES

This article has been awarded <Open Data, Open Materials> Badges. All materials and data are publicly accessible via the Open Science Framework at https://doi.org/10.5061/dryad.76hdr7svb.

## Data Availability

Code of simulation and data are available at Dryad Dryad DOI: https://doi.org/10.5061/dryad.76hdr7svb

## 1

**TABLE A1:** Results of paired *t*‐tests comparing mean values of learning abilities (*L*) of residents and dispersers

Season length (*S*)	*t*	*df*	*p*‐value	Mean *L* resident	Mean *L* disperser
4	7.5912	9	3.36E−05	0.1933035	0.04778159
10	8.5109	9	1.35E−05	0.1767255	0.04029792
20	81.259	9	3.28E−14	0.9840266	0.1138525
30	63.587	9	2.97E−13	0.9704485	0.1546901
50	−1.3462	9	0.2112	0.91882	0.9261572
100	−16.464	9	5.01E−08	0.8181473	0.890475
300	−11.524	9	1.09E−06	0.6514239	0.7353187
500	−5.6239	9	0.0003242	0.5903464	0.6647682
1,000	−5.0862	9	0.0006573	0.5188527	0.5776454
2,000	−8.6489	9	1.18E−05	0.4399097	0.5263899
4,000	−4.7635	9	0.001025	0.3893362	0.4423997

Note

Separately for each season length (*S* = 4 to 4,000) the mean values of *L* of residents and dispersers of each replicated simulation (10 runs per *S*) were used as datapoints.
